# Medial Unicompartmental Knee Arthroplasty Combined With Anterior Cruciate Ligament Reconstruction Yields Similar Outcomes Compared to Unicompartmental Knee Arthroplasty Alone

**DOI:** 10.1155/tsm2/7606835

**Published:** 2025-02-14

**Authors:** Claudio Legnani, Emanuele Massaro, Giuseppe M. Peretti, Vittorio Macchi, Enrico Borgo, Alberto Ventura

**Affiliations:** ^1^Sports Traumatology and Minimally Invasive Articular Surgery Center, IRCCS Orthopedic Institute Galeazzi, Milan, Italy; ^2^Residency Program in Orthopedics and Traumatology, University of Milan, Milan, Italy; ^3^E.U.O.R.R. Unit, IRCCS Orthopedic Institute Galeazzi, Milan, Italy; ^4^Department of Biomedical Sciences for Health, University of Milan, Milan, Italy

**Keywords:** ACL reconstruction, anterior cruciate ligament, knee, unicompartmental knee arthroplasty

## Abstract

**Background:** The treatment of unicompartmental knee osteoarthritis (OA) in young, active individuals with anterior cruciate ligament (ACL) insufficiency is a debatable topic. The objective, radiological, and functional results of medial unicompartmental knee arthroplasty (UKA) combined to ACL reconstruction and those of isolated UKA are compared in the present study.

**Methods:** Twelve patients with medial OA and ACL incompetence were suitable for combined UKA and ACL reconstruction (Group A). A control group consisted of 24 patients who underwent isolated UKA within the same time frame and were matched for age, body mass index, and male/female ratio (Group B). The Oxford Knee Score (OKS), the Knee OA Outcome Score (KOOS), the WOMAC index of OA, and standard X-rays were used for clinical and radiologic evaluation.

**Results:** The mean KOOS score, OKS, and WOMAC index improved 10 years after surgery, demonstrating a statistically significant change (*p* < 0.001). At follow-up, there was no significant between-groups difference concerning KOOS, OKS, or WOMAC scores (*p*=*n*.*s*.). One female patient in Group A underwent revision total knee arthroplasty (TKA) 3 years after the first surgery because OA in the lateral compartment had developed and the patient's discomfort persisted. There were no signs of pathologic radiolucent lines or radiographic signs of implant loosening at the most recent follow-up, which occurred at an average of 7.9 years for Group A and 9.1 years for Group B.

**Conclusion:** Ten years after surgery, UKA combined to ACL reconstruction provides clinical and radiographic results similar to UKA without increasing the incidence of complications.

## 1. Introduction

Unicompartmental knee arthroplasty (UKA) is a valid and reliable surgical strategy for treating advanced isolated medial femorotibial osteoarthritis (OA) [1], which leads to improved clinical outcomes compared to total knee replacement, especially in patients willing to return to active daily living [2, 3].

However, UKA is traditionally not indicated when simultaneous anterior cruciate ligament (ACL) incompetence is present [4], and in this scenario, a surgical method allowing for both UKA and ACL restoration has been recently presented [5–12]. Overall, young and active individuals with unicompartmental knee OA benefited with UKA in combination with additional surgery [13].

Although this surgical technique possesses disadvantages related to its technical difficulties which may determine impingement with the neoligament and potential undersizing of the tibial component, good midterm outcomes have been documented for this combined surgical treatment. In fact, the high risk of problems noted in certain research might prevent this treatment alternative from becoming widely used [12].

The functional, radiological, and subjective results of isolated UKA versus combined medial UKA with ACL reconstruction were compared in this study. Combining the two therapies was thought to produce comparable outcomes without increasing the risk of postoperative complications.

## 2. Patients and Methods

### 2.1. Study Design

Twelve patients with medial OA and ACL incompetence had simultaneous UKA and ACL reconstruction from 2006 to 2010 and were successfully followed up 10 years later (Group A). A retrospective analysis of prospectively collected data from senior's author database was conducted. Patients were matched for age, gender, and body mass index (BMI) in a 1:2 ratio to a control group of 24 patients who got UKA for isolated medial OA and had the same average follow-up period (10.0 years, Group B). The following exclusion criteria applied: BMI greater than 30, multicompartmental OA, over 10° of varus knee deformity and/or tibial slope, and the existence of issues affecting the patellofemoral joint.

Every surgery was performed by a single surgeon. Written informed consent was acquired. Strengthening the Reporting of Observational Studies in Epidemiology (STROBE) principles were followed in conducting the study, as well as guidelines from the Institution's Ethical Committee. Information about radiological and clinical evaluation up to 10 years following surgery was taken from institutional registries. The latest follow-up was taken into account. Since the authors' institution routinely follows up on patient-reported outcomes, the follow-up of this observational analysis employing a retrospective design on well-established surgical procedures did not require ethical approval.

### 2.2. Surgical Procedure and Rehabilitation Protocol

Surgery for UKA combined with ACL reconstruction was conducted as previously described [7] using autologous hamstring tendon grafts fixed proximally with either a RetroButton device (Arthrex, Naples, and FL) or a RIGID-FIX equipment (DePuy Mitek, Raynham, MA, USA) and an Allegretto Unicondylar fixed-bearing prosthesis (Zimmer-Biomet Orthopedics, Warsaw, IN, USA). A BioRCI screw (Bioabsorbable Rounded Cannulated Interference, Smith & Nephew Inc. Andover, MA, USA) was used to perform the distal fixation.

Group B patients were all given a cemented Oxford Partial Knee Arthroplasty (Zimmer-Biomet Orthopedics) by means of a medial parapatellar arthrotomy. A brace-free postoperative rehabilitation plan was started the day after surgery including gradual weight-bearing ambulation with crutches, joint mobility exercises, and an early recovery to full knee extension. Walking with partial weight bearing was allowed for the first 4 weeks with two crutches.

### 2.3. Follow-Up Assessment

The evaluation comprised the Western Ontario and McMaster (WOMAC) index of OA, the Oxford Knee Score (OKS), and the Knee OA Outcome Score (KOOS). Standard radiographs were used for radiological assessment in order to gather data regarding any component loosening that may have occurred. Implant survival was based on performed revision surgery.

### 2.4. Statistical Analysis

The retrieved data were analyzed using SPSS Statistics for Windows, Version 21.0 (IBM Corp., Armonk, NY). The state of follow-up and preoperative care, and any variations between the two groups, was compared using the Wilcoxon signed-rank test. Differences were considered statistically significant with *p* value < 0.05.

## 3. Results

The mean age at surgery was 54 years (SD: 3.9) for Group A and 54.7 years (SD: 3.9) for Group B (Table 1).

The mean overall KOOS score, OKS, and WOMAC index all increased postoperatively, indicating a statistically significant improvement (*p* < 0.001, Table 2).

On the baseline questionnaires, Group A performed worse, although the difference was not statistically significant. Regarding KOOS, OKS, and WOMAC scores, there was no discernible difference between the groups at the most recent follow-up (*p*=*n*.*s*., Table 3).

For Group A, the average radiographic follow-up was 7.9 years (SD: 2.6), whereas Group B had an average of 9.2 years (SD: 1.9). There were no reports of significant side effects such as thrombosis, infections, fractures, or neurovascular injuries. Three years following surgery, a female patient in Group A underwent a revision total knee arthroplasty (TKA) with symptom alleviation due to persistent discomfort and the development of OA in the lateral compartment. One patient in Group B had superficial wound dehiscence, while another patient receiving isolated UKA had persistent anterior knee pain. Therefore, the implant survival rate was 83% for Group A and 100% for Group B at the latest follow-up. There were no radiographic signs of pathologic radiolucent lines or implant loosening at the most recent follow-up in both groups.

## 4. Discussion

The primary result of the present investigation is that UKA in conjunction with ACL reconstruction yields clinical and radiographic results that are equivalent to UKA a decade following surgery, without posing an elevated risk of complications.

Both treatments improve postoperative clinical and functional outcomes throughout the medium to long term. Regarding functional outcomes, at the follow-up, there were no statistically significant differences between the two groups.

UKA is recommended in the presence of a functional ACL. In fact, when medial UKA is performed, the integrity of the ACL provides protection to the prosthetic implant. The tibia's anterior translation is by the ACL. Additionally, it lessens the strain on the polyethylene insert and lateral compartment to some extent [4]. For these reasons, combined UKA and ACL reconstruction has been proposed as a viable treatment option for the ACL-deficient knee with end-stage medial OA [5–12].

According to recent systematic reviews, when performed in the same sitting, ACL reconstruction and UKA can significantly improve functional and clinical results for patients with ACL injuries who present with isolated medial compartment pain and knee instability [14, 15].

Following a mean follow-up of 45 months, 10 patients had hamstring ACL surgery in conjunction with robotically assisted UKA, as reported by Foissey et al., average Tegner score was 4.5, and the average International Knee postoperative function score was 93 [8].

Twenty-three patients were followed up by Jaber et al. for 10 years after mobile-bearing UKA in conjunction with ACL surgery [10]. Mean OKS and Lysholm scores were 40 and 85.5, respectively. All of the patients resumed their physical and athletic activities, with none reporting knee instability. At 14.5 years, survival rate was 91.4%. As in our case series, a patient's symptomatic lateral knee OA progressed following surgery, prompting a conversion procedure 1 year later.

Four years following surgery, the average OKS reported by Tian et al. on 28 patients treated who had undergone combined UKA and ACL reconstruction was 43, and the mean Tegner activity level was 5.3 [5]. In the Kurien et al. trial, medial UKA and ACL surgery were performed simultaneously on 24 patients. Five years after surgery, a mean Lysholm score of 92 and OKS score of 46 were reported. Fixed-bearing UKA was selected in accordance with our findings in order to reduce the possibility of bearing dislocation [9, 16].

Our results revealed no statistically significant difference in the two groups' KOOS, OKS, or WOMAC ratings 10 years after surgery. This shows that equivalent results to UKA can be obtained with combined UKA and ACL reconstruction. This suggests that treating relatively young, active individuals with advanced medial knee OA and ACL insufficiency with this combined procedure is successful.

The technically difficult nature of this combined operation, as well as the possibility of postoperative stiffness, graft impingement, and tibial component undersizing, is its drawbacks. Combined ACL reconstruction and UKA should be favored over a phased surgical procedure to lessen these drawbacks. Graft impingement of the neoligament can actually be avoided by drilling the tibial tunnel aperture more vertically and leaving it laterally [8].

Given the strict selection criteria needed for the procedure, our study possesses inevitable limitations: the relatively small study sample and the lack of a power analysis, given the retrospective nature of the study design, may have prevented the identification of subtle group differences. The findings of the surgery indicated a preference for isolated UKA, although not being supported by statistical significance. Additional limitations were that ACL reconstruction results were not evaluated through clinical or radiographic examination, nor instrumented laxity measurement. Furthermore, the study included only patients with a BMI < 30; therefore, our findings may not be generalizable to obese patients. It is necessary to conduct multicenter researches encompassing a larger number of patients in order to enhance the validity of the findings.

## 5. Conclusion

ACL reconstruction combined with UKA is a clinically feasible therapy option for young, active individuals who have had an initial ACL injury and develop secondary OA. Up to 10 years after surgery, it validates both subjective and objective clinical improvement, with radiographic and clinical results matching those of unilateral UKA.

## Figures and Tables

**Table 1:** Patient demographics and anthropometric data.

	UKA + ACL reconstruction	UKA
No. of patients	12	24
Gender		
Male	8	16
Female	4	8
Mean age at surgery (range) (yr)	54.0 (3.9)	54.7 (3.9)
Mean BMI (range)	25.9 (2.4)	26.0 (1.6)

Abbreviations: ACL, anterior cruciate ligament; BMI, body mass index; SD, standard deviation; UKA, unicompartmental knee arthroplasty.

**Table 2:** Comparison between preoperative and follow-up status.

	UKA + ACL reconstruction			UKA		
	Preoperative	Follow-up	*p* value	Preoperative	Follow-up	*p* value
KOOS (mean, SD)	62.4 (8.1)	80.1 (9.8)	< 0.001	61.5 (9.3)	81.1 (5.4)	< 0.001
OKS (mean, SD)	28.8 (10.1)	42.6 (9.0)	< 0.001	29.8 (5.9)	43.3 (4.3)	< 0.001
WOMAC score (mean, SD)	71.9 (11.5)	84.6 (8.8)	< 0.001	69.5 (4.6)	85.0 (3.0)	< 0.001

*Note:* WOMAC: Western Ontario and McMaster University Osteoarthritis Index.

Abbreviations: ACL, anterior cruciate ligament; KOOS, Knee Osteoarthritis Outcome Score; OKS, Oxford Knee Score; SD, standard deviation; UKA, unicompartmental knee arthroplasty.

**Table 3:** Comparison between groups at final follow-up.

	UKA + ACL reconstruction	UKA	*p* value
KOOS (mean, SD)	80.1 (9.8)	81.1 (5.4)	0.68
OKS (mean, SD)	42.6 (9.0)	43.3 (4.3)	0.73
WOMAC score (mean, SD)	84.6 (8.8)	85.0 (3.0)	0.83

*Note:* WOMAC: Western Ontario and McMaster University Osteoarthritis Index.

Abbreviations: ACL, anterior cruciate ligament; KOOS, Knee Osteoarthritis Outcome score; OKS, Oxford Knee Score; SD, standard deviation; UKA, unicompartmental knee arthroplasty.

## Data Availability

Data are available upon reasonable request.

## References

[1] Neubauer M., Reinberger E. M., Dammerer D. (2023). Unicompartmental Knee Arthroplasty Provides Superior Clinical and Radiological Outcomes Compared to High Tibial Osteotomy at a Follow-Up of 5-8 Years. *Journal of Clinical Medicine*.

[2] Ventura A., Macchi V., Borgo E., Legnani C. (2022). Shift to Low-Impact Sports and Recreational Activities Following Total Knee Replacement. *The International Journal of Artificial Organs*.

[3] Legnani C., Macchi V., Peretti G. M., Coccioli G., Borgo E., Ventura A. (2023). Return to Sports and Physical Activities Following Mobile-Bearing Unicompartmental Knee Replacement. *Journal of Biological Regulators & Homeostatic Agents*.

[4] Williams R. J. I. I. I., Wickiewicz T. L., Warren R. F. (2000). Management of Unicompartmental Arthritis in the Anterior Cruciate Ligament Deficient Knee. *The American Journal of Sports Medicine*.

[5] Tian S., Wang B., Wang Y., Ha C., Liu L., Sun K. (2016). Combined Unicompartmental Knee Arthroplasty and Anterior Cruciate Ligament Reconstruction in Knees with Osteoarthritis and Deficient Anterior Cruciate Ligament. *BMC Musculoskeletal Disorders*.

[6] Tecame A., Savica R., Rosa M. A., Adravanti P. (2019). Anterior Cruciate Ligament Reconstruction in Association With Medial Unicompartmental Knee Replacement: A Retrospective Study Comparing Clinical and Radiological Outcomes of Two Different Implant Design. *International Orthopaedics*.

[7] Ventura A., Legnani C., Terzaghi C., Macchi V., Borgo E. (2020). Unicompartmental Knee Replacement Combined to Anterior Cruciate Ligament Reconstruction: Midterm Results. *Journal of Knee Surgery*.

[8] Foissey C., Batailler C., Shatrov J., Servien E., Lustig S. (2023). Is Combined Robotically Assisted Unicompartmental Knee Arthroplasty and Anterior Cruciate Ligament Reconstruction a Good Solution for the Young Arthritic Knee?. *International Orthopaedics*.

[9] Kurien T., Stragier B., Senevirathna S., Geutjens G. (2022). Excellent Outcomes With Combined Single Stage Physica ZUK Medial Unicompartment Knee Replacement and Anterior Cruciate Ligament Reconstruction Results in Young, Active Patients With Instability and Osteoarthritis With a Mean Follow up of 5 Years. *The Knee*.

[10] Jaber A., Kim C. M., Barié A. (2023). Combined Treatment With Medial Unicompartmental Knee Arthroplasty and Anterior Cruciate Ligament Reconstruction Is Effective on Long-Term Follow-Up. *Knee Surgery, Sports Traumatology, Arthroscopy*.

[11] Legnani C., Borgo E., Macchi V., Terzaghi C., Ventura A. (2024). Unicompartmental Knee Replacement Combined With Anterior Cruciate Ligament Reconstruction Provides Comparable Results to Total Knee Replacement With No Increased Risk of Complications. *SICOT-J.*.

[12] Iriberri I., Suau S., Payán L., Aragón J. F. (2019). Long-term Deterioration After One-Stage Unicompartmental Knee Arthroplasty and Anterior Cruciate Ligament Reconstruction. *Musculoskelet Surg*.

[13] Derreveaux V., Schmidt A., Shatrov J. (2022). Combined Procedures With Unicompartmental Knee Arthroplasty: High Risk of Stiffness but Promising Concept in Selected Indications. *SICOT J*.

[14] Legnani C., Muzzi S., Peretti G. M., Borgo E., Ventura A. (2021). Anterior Cruciate Ligament Reconstruction Combined to Partial Knee Replacement in Active Patients With ACL Deficiency and Knee Osteoarthritis. *The Physician and Sportsmedicine*.

[15] Albo E., Campi S., Zampogna B. (2021). Results of Simultaneous Unicompartmental Knee Arthroplasty and Anterior Cruciate Ligament Reconstruction: A Systematic Review. *Journal of Clinical Medicine*.

[16] Legnani C., Ventura A., Mangiavini L., Maffulli N., Peretti G. M. (2024). Management of Medial Femorotibial Knee Osteoarthritis in Conjunction with Anterior Cruciate Ligament Deficiency: Technical Note and Literature Review. *Journal of Clinical Medicine*.

